# Expression, purification and crystallization of a novel metagenome-derived salicylaldehyde dehydrogenase from Alpine soil

**DOI:** 10.1107/S2053230X22002345

**Published:** 2022-03-28

**Authors:** Shamsudeen Umar Dandare, Maria Håkansson, L. Anders Svensson, David J. Timson, Christopher C. R. Allen

**Affiliations:** a School of Biological Sciences, Queen’s University Belfast, 19 Chlorine Gardens, Belfast BT9 5DL, United Kingdom; b School of Natural and Built Environment, Queen’s University Belfast, David Kier Building, Stranmillis Road, Belfast BT9 5AG, United Kingdom; c SARomics Biostructures AB, Medicon Village, 223 81 Lund, Sweden; d School of Pharmacy and Biomolecular Sciences, University of Brighton, Huxley Building, Lewes Road, Brighton BN2 4GJ, United Kingdom; e Institute for Global Food Security, Queen’s University Belfast, 19 Chlorine Gardens, Belfast BT9 5DL, United Kingdom

**Keywords:** metagenome, salicylaldehyde dehydrogenase, alphaproteobacteria, Alpine soil, purification, crystallography

## Abstract

Salicylaldehyde dehydrogenase (SALD) catalyses the last reaction in the upper pathway of naphthalene degradation: the oxidation of salicylaldehyde to salicylate. This enzyme has been isolated and studied from a few organisms that belong to the betaproteobacteria and gammaproteobacteria, predominantly *Pseudomonas putida*. Furthermore, there is only one crystal structure of this enzyme, which was obtained from *P. putida* G7. Here, crystallographic studies and analysis of the crystal structure of an Alpine soil metagenome-derived SALD (SALD_AP_) from an alphaproteobacterium are presented. The SALD_AP_ gene was discovered using gene-targeted sequence assembly and it was cloned into a pLATE51 vector. The recombinant protein was overexpressed in *Escherichia coli* BL21 (DE3) cells and the soluble protein was purified to homogeneity. The protein crystallized at 20°C and diffraction data from the crystals were collected at a resolution of 1.9 Å. The crystal belonged to the orthorhombic space group *C*222_1_, with unit-cell parameters *a* = 116.8, *b* = 121.7, *c* = 318.0 Å. Analysis of the crystal structure revealed its conformation to be similar to the organization of the aldehyde dehydrogenase superfamily with three domains: the catalytic, NAD^+^-binding and bridging domains. The crystal structure of NahF from *P. putida* G7 was found to be the best structural homologue of SALD_AP_, even though the enzymes share only 48% amino-acid identity. Interestingly, a carboxylic acid (protocatechuic acid) was found to be a putative ligand of the enzyme and differential scanning fluorimetry was employed to confirm ligand binding. These findings open up the possibility of studying the mechanism(s) of product inhibition and biocatalysis of carboxylic acids using this enzyme and other related aldehyde dehydrogenases.

## Abstract 2

Gene-targeted assembly was used to mine a novel salicylaldehyde dehydrogenase from an Alpine soil metagenome. The enzyme was cloned, expressed, purified and crystallized: it is the first metagenome-derived aldehyde dehydrogenase to be crystallized. Analysis of the crystal structure shows that it adopts the standard conformation of the aldehyde dehydrogenase superfamily and a carboxylic acid was found to be a putative ligand of this enzyme.

## 1. Introduction

Polycyclic aromatic hydrocarbons (PAHs) are aromatic pollutants that are recalcitrant to degradation and therefore tend to accumulate in the ecosystem. These aromatic compounds consist of multiple fused rings, with the most common ones being five- or six-membered rings, which include anthracene, benzo[a]pyrene, naphthalene, phenanthrene and pyrene (Wang *et al.*, 2017 ▸). These hydrophobic compounds are ubiquitous in the environment and pose serious health hazards since they are toxic, teratogenic, carcinogenic and mutagenic (Lee *et al.*, 2018 ▸; Dastgheib *et al.*, 2012 ▸).

During the degradation of various aromatic hydrocarbons several metabolic intermediates are found, which include some aromatic aldehydes and their derivatives. Noteworthy is salicylaldehyde, which is a key intermediate in naphthalene, phenanthrene, acenaphthene and carbaryl degradation pathways (Ghosal *et al.*, 2016 ▸; Mallick *et al.*, 2011 ▸). Aldehydes are also vital intermediates in the metabolism of macromolecules and xenobiotics. Although aromatic and aliphatic aldehydes are extensively used in industry, these compounds have been found to be toxic to life (Caboni *et al.*, 2013 ▸; Roy & Das, 2010 ▸).

As a key intermediate in the breakdown of some aromatic PAHs, salicylaldehyde can be oxidized to salicylate by the activity of salicylaldehyde dehydrogenase (SALD; EC 1.2.1.65), which is an NAD(P)^+^-dependent enzyme. In naphthalene degradation, this enzyme catalyses the last reaction of the upper pathway (Seo *et al.*, 2009 ▸; Eaton & Chapman, 1992 ▸). SALD belongs to the superfamily of NAD(P)^+^-dependent aldehyde dehydrogenases (ALDHs). Generally, the enzymes of this superfamily catalyse the oxidation of a broad range of aldehydes to their corresponding carboxylic acids, playing a major role in detoxification. Structurally, the scaffold of ALDHs is comparable, in which they possess three domains: an NAD(P)^+^ cofactor-binding domain, a catalytic domain and a bridging domain (Marchler-Bauer *et al.*, 2013 ▸; Perozich *et al.*, 1999 ▸).

Several studies have reported the *in vivo* activity of SALDs from a range of aromatic hydrocarbon-degrading microorganisms (Rosselló-Mora *et al.*, 1994 ▸; Grund *et al.*, 1992 ▸; Schell, 1983 ▸), and a few studies have described the purification and characterization of the enzyme (Coitinho *et al.*, 2016 ▸; Singh *et al.*, 2014 ▸). Only Coitinho *et al.* (2016 ▸) have reported a crystal structure of this enzyme, which they isolated from *Pseudomonas putida* G7.

In our laboratory, we have recently been engaged in the discovery and characterization of novel enzymes from Alpine metagenomes (Dandare *et al.*, 2019 ▸). Here, we report the exploitation of molecular-biology strategies (cloning, heterologous overexpression and protein purification) to obtain the novel Alpine metagenome-derived SALD_AP_. We further crystallized the enzyme, collected diffraction data and solved its structure. To the best of our knowledge, this is the first report of the crystallization and structure of a metagenome-derived ALDH.

## 2. Materials and methods

### 2.1. Macromolecule production

#### 2.1.1. Molecular cloning

Following the discovery of SALD_AP_ by gene-targeted assembly, DNA was isolated from Alpine soil samples (Young *et al.*, 2019 ▸) and used as the template for polymerase chain reaction (PCR) to amplify the SALD_AP_ gene. The primers, expression vector and host are given in Table 1 ▸.

The DNA fragments obtained on a 1% agarose gel after PCR were excised, purified and inserted into a pLATE51 vector (Thermo Fisher Scientific). The resulting recombinant p51-SALD_AP_ plasmid was then transformed into *Escherichia coli* BL21 (DE3) chemically competent cells and positive transformants were confirmed by colony PCR and sequencing using the pLATE vector primers.

#### 2.1.2. Protein expression

A confirmed positive clone was inoculated in 10 ml LB broth supplemented with 100 µg ml^−1^ ampicillin and grown overnight. A 2 l flask containing 800 ml LB broth supplemented with 100 µg ml^−1^ ampicillin was then inoculated with 5 ml of the overnight culture of the recombinant p51-SALD_AP_ cells. The large-scale culture was incubated at 30°C with shaking (200 rev min^−1^) until the mid-exponential phase of growth (OD_600_ ≃ 0.6); it was then induced for protein expression with a final concentration of 1 m*M* isopropyl β-d-1-thiogalactopyranoside and allowed to grow under the same conditions for 6 h.

The bacterial cells were harvested by centrifugation (4°C, 7000*g*, 30 min) and resuspended in 20 ml lysis buffer (50 m*M* NaH_2_PO_4_, 300 m*M* NaCl, 5 m*M* imidazole pH 8.0, 0.2 mg ml^−1^ lysozyme, 0.5 m*M* phenylmethylsulfonyl fluoride). Cell lysis was achieved by mechanical disruption using a Soniprep 150 with three successive cycles at an amplitude of 16 µm. Each sonication cycle included 30 s on per pulse on an ice bath to minimize heat accumulation, which could consequently lead to protein degradation. Subsequently, the supernatant containing the soluble protein was separated from the cell debris by centrifugation at 4°C for 30 min at 15 000*g*.

#### 2.1.3. Purification

The expressed protein possessed an N-terminal 6×His tag; therefore, it was purified by metal-affinity chromatography using HIS-Select cobalt (Co^2+^) affinity resin. An Econo column was packed with 1 ml of the resuspended resin and equilibrated with four column volumes of equilibration buffer (50 m*M* NaH_2_PO_4_, 300 m*M* NaCl, 10 m*M* imidazole pH 8.0). The supernatant containing the recombinant protein was poured into the column and the flowthrough was collected. The column was washed twice in each cycle with four column volumes of equilibration buffer. Finally, 4 ml of elution buffer (50 m*M* NaH_2_PO_4_, 300 m*M* NaCl, 250 m*M* imidazole pH 8.0) containing a high concentration of imidazole was used to elute the retained His-tagged protein from the affinity resin.

Subsequently, the eluted recombinant 6×His SALD_AP_ protein was extensively dialysed against dialysis buffer (20 m*M* sodium phosphate buffer pH 7.5, 20 m*M* NaCl). The dialysed protein was further purified using gel filtration on a K 9/30 chromatography column prepacked with Sephacryl S-300 (Pharmacia) with a bed volume (*V*_t_) of 48 ml. Prior to sample loading, the column was equilibrated with gel-filtration buffer (50 m*M* Tris–HCl, 17 m*M* Tris base, 150 m*M* NaCl pH 7.4) at a flow rate of 1 ml min^−1^. The void volume (*V*_0_) was determined using blue dextran, and the column was then calibrated with standard proteins (β-amylase, 200 kDa; bovine serum albumin, 66 kDa; carbonic anhydrase, 29 kDa; cytochrome *c*, 12.4 kDa), which were used to plot a standard curve in order to assess the oligomerization state of SALD_AP_. Approximately 300 µl of protein sample was loaded onto the column and 1 ml fractions were collected. The presence of protein in each fraction was determined by measuring the absorbance at 280 nm using a 6705 UV–visible spectrophotometer. The fractions with the highest absorbance were pooled and concentrated using an Amicon Ultra-30k centrifugal filter (Millipore) until a final protein concentration of 12 mg ml^−1^ was obtained, which was used in crystallization trials.

### 2.2. Crystallization

SALD_AP_ at a concentration of 12 mg ml^−1^ in 20 m*M* sodium phosphate buffer, 20 m*M* NaCl pH 7.4 was used in crystallization experiments with 2 m*M* tris(2-carboxyethyl)phosphine (TCEP) added to keep the protein reduced. Before crystallization experiments, the protein solution was centrifuged at 10 000*g* at 4°C for 10 min. The crystal was grown from the JCSG+ screen (Molecular Dimensions) at 20°C in a 100 + 100 nl drop set up over 40 µl reservoir consisting of 0.1 *M* sodium acetate pH 4.6, 8%(*w*/*v*) PEG 8000. Table 2 ▸ shows a summary of the experimental crystallization setup.

**Figure 1:**
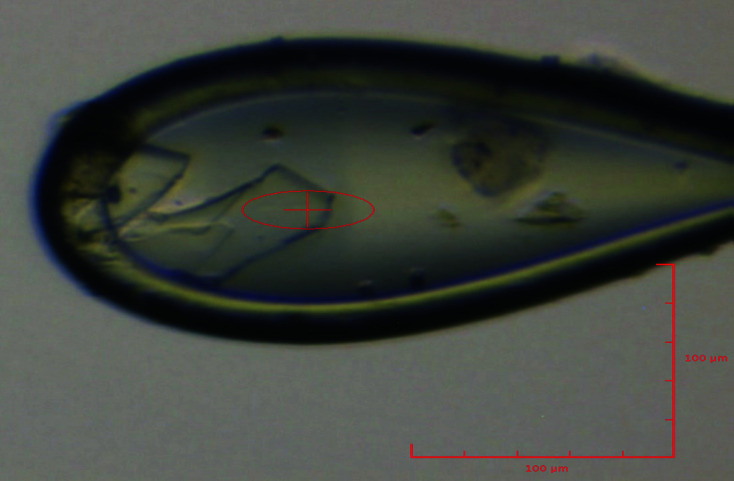
The crystal mounted in a nylon loop in the X-ray beam. The red cross indicates the position of the X-ray beam. The red bars indicate the scale and correspond to 100 × 100 µm.

Before flash-cooling in liquid nitrogen, the crystal was dipped into cryosolution consisting of 0.1 *M* sodium acetate pH 4.6, 10%(*w*/*v*) PEG 8000, 25%(*v*/*v*) glycerol, 2 m*M* TCEP. Data were collected from a fragment of a crystal with an original size of about 60 × 40 × 10 µm, as seen in Fig. 1 ▸.

### 2.3. Data collection and processing

Data were collected to 1.9 Å resolution at 100 K on beamline I03 (λ = 1.03865 Å) at Diamond Light Source using a PILATUS3 6M detector (Dectris). Data were processed using *XDS* (Kabsch, 2010 ▸) and *AIMLESS* (Evans & Murshudov, 2013 ▸). For *R*_free_ calculations, 5% of the reflections were flagged and were not used for structure refinement.

### 2.4. Structure solution and refinement

The structure was determined with *Phaser* (McCoy *et al.*, 2007 ▸) using a modified model of PDB entry 4jz6 (salicyl­aldehyde dehydrogenase from *P. putida* G7 complexed with salicylaldehyde; Coitinho *et al.*, 2016 ▸) as a starting model (48% identity to the SALD_AP_ amino-acid sequence). Thereafter, the model was rebuilt using the molecular-graphics software *Coot* (Emsley *et al.*, 2010 ▸) and refined using the reciprocal-space refinement program *REFMAC*5 (Murshudov *et al.*, 2011 ▸) with riding hydrogen atoms, noncrystallographic symmetry (NCS) restraints between the four independent molecules and TLS parametrization (Winn *et al.*, 2001 ▸). In the final stages of refinement, *BUSTER* (Bricogne *et al.*, 2016 ▸) was used for refinement. Several ligands were tested and the ligand that best fitted the electron density was used in the final stages of refinement.

### 2.5. Homologous structural comparison

In order to identify proteins that are structurally similar to SALD_AP_, a protein structure-comparison server (the *DALI* server; http://ekhidna2.biocenter.helsinki.fi/dali/) was used to perform a three-dimensional search (Holm & Rosenström, 2010 ▸). Further structural comparisons and analysis of SALD_AP_ and the best structural homologue were carried out by superimposition of the crystal structures using *PyMOL* version 1.7.4.5.

### 2.6. Differential scanning fluorimetry (DSF)

The thermal stability of the enzyme with and without its ligand(s) was determined using DSF. Initially, the optimum enzyme concentration that gave the best fluorescence signal was determined by enzyme titration (5–7 µ*M*). Also, the optimum concentration of ligand that gave the best signal was determined by measuring different concentrations (0.5–2.0 m*M*). The assay mixture was made up to a final volume of 20 µl consisting of the enzyme aliquot diluted to the appropriate concentration in 50 m*M* HEPES pH 7.4. Ligands and cofactor (NAD^+^) were added where required. SYPRO Orange (1× working concentration) was always the last component to be added to the reaction mixture prior to running in the thermocycler. All reactions were prepared on ice to minimize protein denaturation.

The reactions were prepared in 0.2 ml PCR tubes in triplicate and were run in a Rotor-Gene Q cycler (Qiagen). A high-resolution melt experiment with the following protocol was set up: a temperature rise from 25 to 95°C with a 1°C increase every 5 s without gain optimization. The fluorescence of the protein due to the binding of SYPRO (dye) to its exposed hydrophobic regions as it denatures with increasing temperature was exploited by exciting the enzyme at 460 nm and measuring the emission at 510 nm. This assay was used as a measure of the thermal stability of the enzyme in the presence and absence of ligands.

First-derivative (Δ*F*/Δ*T*) plots of the melting curves of the enzyme were used to determine the melting temperature (*T*_m_) of the enzyme. *T*_m_ is the temperature at which the Δ*F*/Δ*T* peak appears. The Rotor-Gene inbuilt analysis software was used to calculate the derivative of fluorescence over temperature and the *T*_m_. The melting temperatures of SALD_AP_ with and without its ligands were determined and compared in order to ascertain its thermal stability.

## 3. Results and discussion

**Figure 2:**
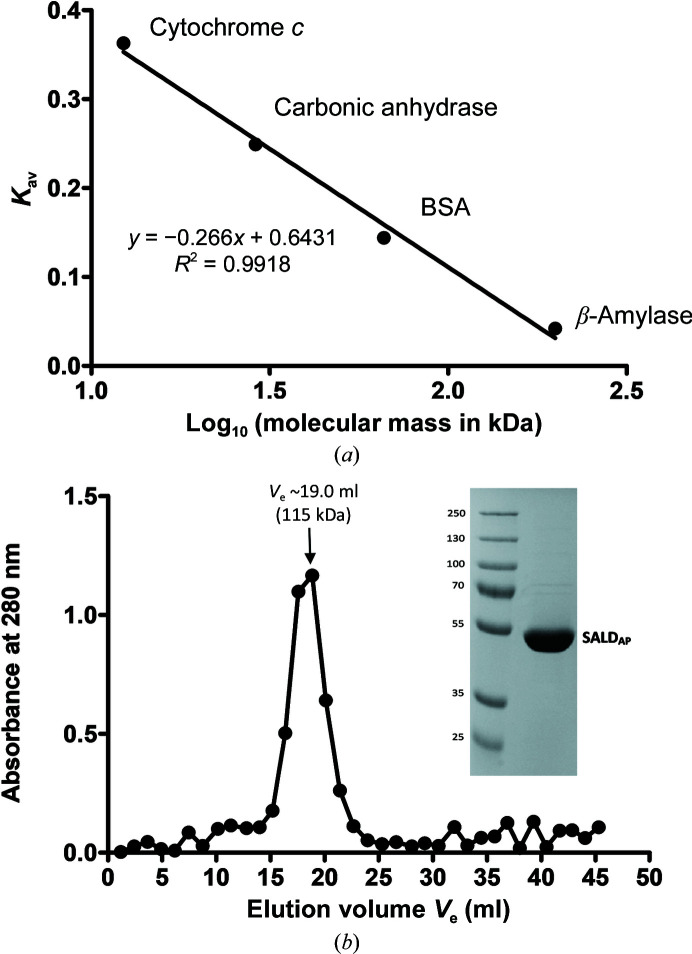
(*a*) The calibration graph for the estimation of protein molecular mass. (*b*) Typical analytical gel-filtration chromatogram obtained with recombinant SALD_AP_. The elution of SALD_AP_ corresponds to the profile of a 115 kDa protein, which is in agreement with a dimeric form of SALD_AP_. The points represent individual absorbance (*A*_280_) readings and the SDS gel shows the eluted fraction corresponding to the highest absorbance.

A recombinant Alpine metagenome-derived salicylaldehyde dehydrogenase (SALD_AP_) was overexpressed in its soluble form in *E. coli* BL21 (DE3) cells and the protein was successfully purified to homogeneity using Co^2+^-affinity and gel-filtration chromatography. The elution profile of the gel-filtration chromatogram suggests that the biological unit of SALD_AP_ is a dimer with a protein molecular mass of 115 kDa (Fig. 2 ▸ *b*). This finding is in good agreement with the theoretical protein molecular mass of 110 kDa. Dimerization is a structural property of class 3 aldehyde dehydrogenases such as vanillin dehydrogenase and benzaldehyde dehydrogenase, and differs from the tetrameric assembly (pair of dimers) of the native conformation of class 1 and 2 ALDHs (Rodriguez-Zavala & Weiner, 2002 ▸). A sequence-identity search in the PDB reveals that SALD_AP_ shares 48% amino-acid identity with its closest homologue, a salicylaldehyde dehydrogenase (NahF) from *P. putida* G7. The structure of NahF (PDB entry 4jz6) was the only available crystal structure of a salicylaldehyde dehydrogenase in the PDB prior to our findings.

The purified 6×His-SALD_AP_ was concentrated to 12 mg ml^−1^ and crystals suitable for diffraction were grown. Diffraction data were collected to 1.9 Å resolution from a fragment of a crystal with an original size of about 60 × 40 × 10 µm. The crystals grew in the orthorhombic space group *C*222_1_, with unit-cell parameters *a* = 116.8, *b* = 121.7, *c* = 318.0 Å, and diffracted to 1.9 Å resolution. A summary of the data statistics is presented in Table 3 ▸.

**Figure 3:**
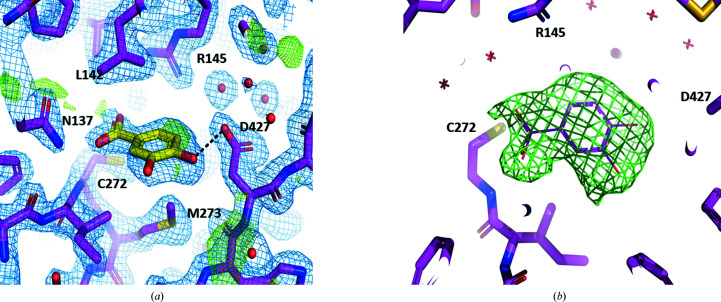
(*a*) The electron density seen in the active site of independent molecule *A* after refinement; there are similar interactions in molecules *B*, *C* and *D* (not shown). The light blue chicken-wire nets are the 2*F*_o_ − *F*_c_ Fourier map with a cutoff of 1σ, while those in green are the *F*_o_ − *F*_c_ difference map at +3σ cutoff and −3σ cutoff. The ligand, PCA/DHB, is drawn with yellow C atoms and salicylaldehyde dehydrogenase residues are drawn with magenta C atoms; water molecules are shown as red spheres. (*b*) A representation of the difference electron-density map (*F*_o_ − *F*_c_) is shown as a green chicken-wire net after refinement of the structure without the ligands of the four independent molecules. The map was drawn at a 3.0σ level at the ligand-binding site of molecule *C* (which shows the highest difference density peak in the difference map). The protein is shown in stick representation, while the position of the ligand in the complex structure is shown in line representation for comparison. A cutoff of 3.5 Å radius around the ligand atoms was used in drawing the difference map.

The final model after refinement includes 470 amino-acid residues in polypeptide chains *A*, *B*, *C* and *D*, with one bound ligand per monomer. A summary of the refinement statistics is presented in Table 4 ▸. Additionally, the model contains one glycerol molecule and 1597 water molecules. The first observed residue is Thr5 and the last is the C-terminal Ile470 in all four chains. No His tags or NAD^+^/NADH were observed in the electron-density maps. Several ligands were tried for refinement, including salicylaldehyde, 2-naphthaldehyde, vanillin and pyrene-1-carboxaldehyde. The latter molecule was too large for the observed ligand density, while the former three molecules all fitted well in the electron density (ED); however, they still showed some residual positive ED in the Fourier map after refinement. Although the EDs were good, protocatechuic acid (PCA) was chosen as the ligand bound to the active site for refinement as it fitted the ED better. A hydrogen bond between the *para*-hydroxyl group of the ligand and the Asp427 side chain is indicated as a black broken line in Fig. 3 ▸(*a*). The electron-density map of the enzyme without the ligand is shown in Fig. 3 ▸(*b*). Interestingly, the binding of a carboxylic acid in the active site of the aldehyde dehydro­genase indicates the potential for product inhibition of the enzyme during aldehyde oxidation; this also means that the enzyme may possess carboxylic acid reductase activity. In the crystal structure of NahF, Coitinho *et al.* (2016 ▸) showed the binding of salicylaldehyde to an invariant cysteine residue that is present in all ALDHs. The mechanism of binding and catalysis of aldehydes in ALDHs has been well studied; however, attention has not been paid to the role of carboxylic acids as ligands of ALDHs. Our finding opens up the possibility of studying the mechanism(s) of product inhibition and potential biocatalysis of carboxylic acids using this enzyme and other related aldehyde dehydrogenases.

**Figure 4:**
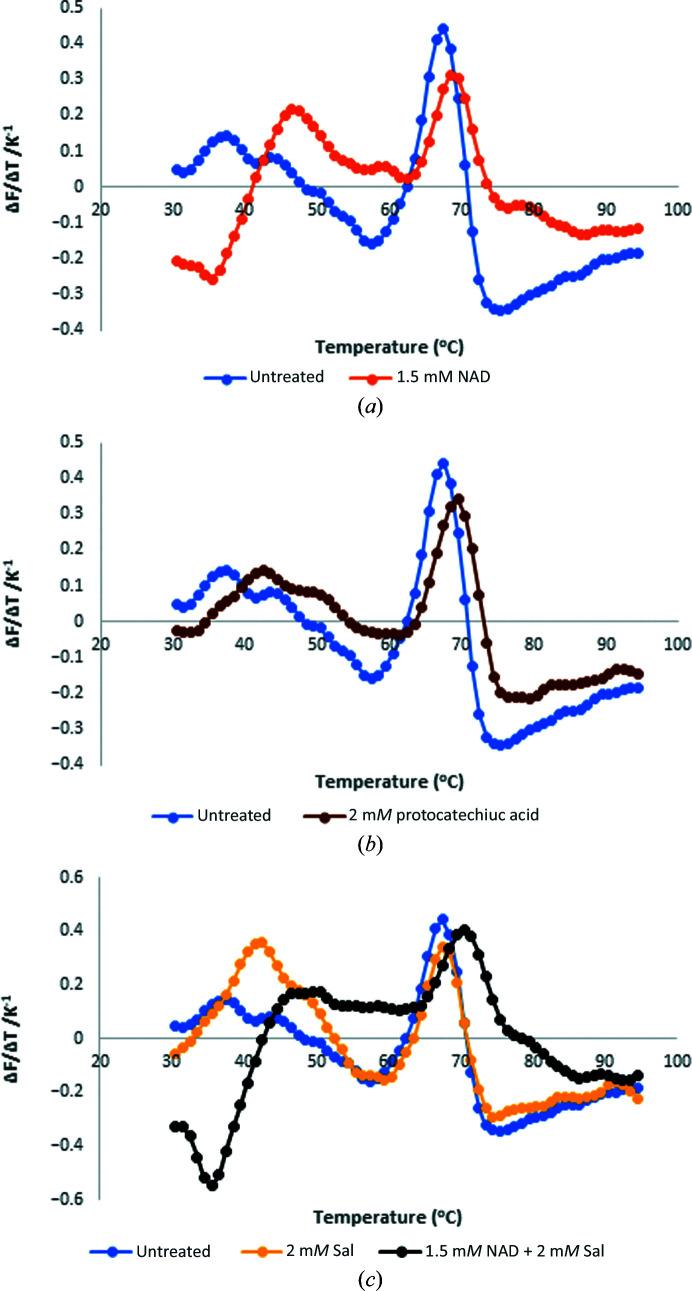
The melting curves of SALD_AP_ showing changes in melting temperature upon binding of the enzyme (*a*) with 1.5 m*M* NAD^+^, (*b*) with 2 m*M* protocatechuic acid and (*c*) with 2 m*M* salicylaldehyde and with a combination of 1.5 m*M* NAD^+^ and 2 m*M* salicylaldehyde.

To prove that PCA is a putative ligand of the enzyme, we carried out differential scanning fluorimetry (DSF) to show that the ligand stabilizes the protein upon binding. DSF shows that SALD_AP_ is thermally stable at ∼68°C, which is higher than the thermostability reported for some yeast ALDHs (Datta *et al.*, 2016 ▸, 2017 ▸). Above 68°C, SALD_AP_ starts to melt and therefore loses its three-dimensional structure, which is required for its activity. In the presence of PCA the melting temperature (*T*_m_) increases to 69.2°C, which reveals that the binding of such a ligand further stabilizes the protein (Fig. 4 ▸). However, further studies such as inhibition and/or biocatalysis with PCA and site-directed mutagenesis need to be carried out to ascertain that PCA is a true ligand of SALD_AP_ and its biological relevance. Because the enzyme is an NAD-dependent salicyl­aldehyde dehydrogenase, we also carried out DSF with NAD^+^ and salicylaldehyde individually and in combination. Interestingly, neither salicylaldehyde nor NAD^+^ exclusively stabilized SALD_AP_. However, significant (*p* < 0.05) stabilization of the protein was observed in the presence of both the cofactor and the substrate, with a *T*_m_ of ∼70°C (Table 5 ▸).

**Figure 5:**
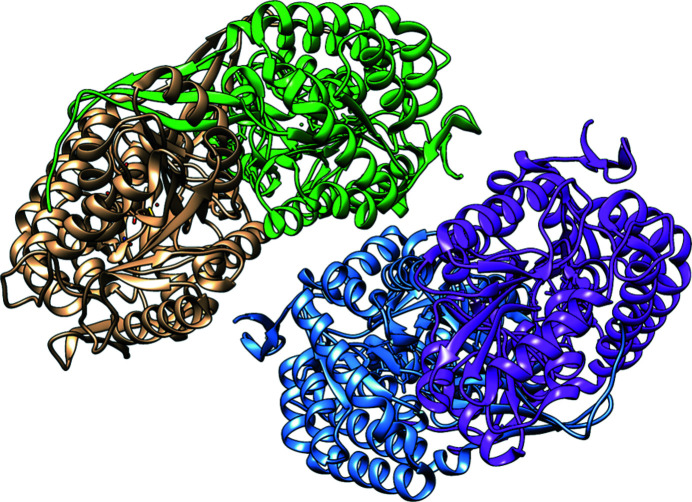
The overall crystal structure of SALD_AP_ shows two homodimers in the asymmetric unit. Chains *A* and *C* forming a dimer are coloured green and gold, respectively, while chains *B* and *D* forming the second dimer are coloured blue and magenta, respectively.

The overall crystal structure of SALD_AP_ shows two independent homodimers in the asymmetric unit (Fig. 5 ▸). Polypeptide chains *A* and *C* formed the first dimer, while chains *B* and *D* formed the second homodimer. The crystallo­graphically independent molecules *B*, *C* and *D* were modelled identically to molecule *A*. The crystallographically independent homodimers further confirm the finding from analytical gel filtration that the biological unit of SALD_AP_ exists as a dimer.

**Figure 6:**
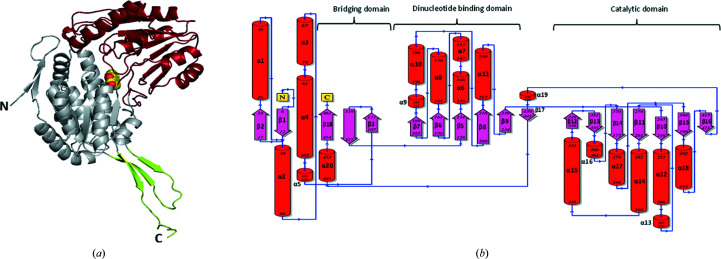
Different representations of the overall fold of novel SALD_AP_ showing (*a*) the monomer as a cartoon model with the N- and C-termini labelled. The catalytic, cofactor-binding and bridging domains are coloured red, grey and lemon, respectively. The C and O atoms of the protocatechuic acid molecule are depicted as yellow and red spheres, respectively. (*b*) Topology diagram. Helices are shown as tubes, while β-strands are shown as arrows; both are labelled numerically. The N- and C-termini are coloured yellow.

SALD_AP_ adopts the standard conformation of the ALDH superfamily. The monomer shows that the enzyme is a classical aldehyde dehydrogenase showing the typical α/β aldehyde dehydrogenase superfamily organization with three domains: catalytic, NAD^+^-binding and bridging domains (Fig. 6 ▸ *a*). The biological unit (dimer) of SALD_AP_ is formed by the oligomerization of two monomers through the bridging domain. The bridging or oligomerization domain is characterized by three β-sheets (β3, β4 and β18) that run antiparallel. The formation of the dimer involves interactions between α-helices α11 (residues 217–231) of each subunit and β-strands of the adjacent subunit (β16, residues 417–420, and β18, residues 455–461) (Fig. 6 ▸ *b*). The oligomerization domain is typical of that found in class 3 ALDHs, with the C-terminal portion of the protein pointing away from a position that favours the interaction of a dimer–dimer interface (tetramer), thus only favouring the formation of a dimer. Rodriguez-Zavala & Weiner (2002 ▸) found a striking difference in both the sequence and the structure of the C-terminal ‘tail’ of ALDH1 and ALDH3, and they demonstrated that the hydrophobic surface area found in this region is the primary force that drives the formation of tetramers. This hydrophobic surface area was found to increase in the tetrameric enzyme (ALDH1) compared with the dimeric ALDH3. The C-terminus of ALDHs was also found to be ultimately involved in the stability of the proteins. The nucleotide-binding domain conforms to the Rossmann fold consisting of five parallel β-strands (β5–β9) connected to six α-helices (α6–α11). Although an NAD^+^ molecule was not found in the cofactor-binding site, the potential residues implicated in the interaction with NAD^+^ adopted a fold quite similar to those observed in other NAD^+^-dependent ALDH complex structures.

Structural comparison of the newly solved SALD_AP_ structure with crystal structures available in the PDB revealed ALDHs with high structural similarity to SALD_AP_. The structural matches were analysed using the PDB90, which is a representative subset of PDB chains in which no two chains share more than 90% sequence identity with each other. Table 6 ▸ shows the first ten homologues of the 124 structures returned by the *DALI* server. The homologues are arranged according to rank, *Z*-score and percentage sequence identity.

**Figure 7:**
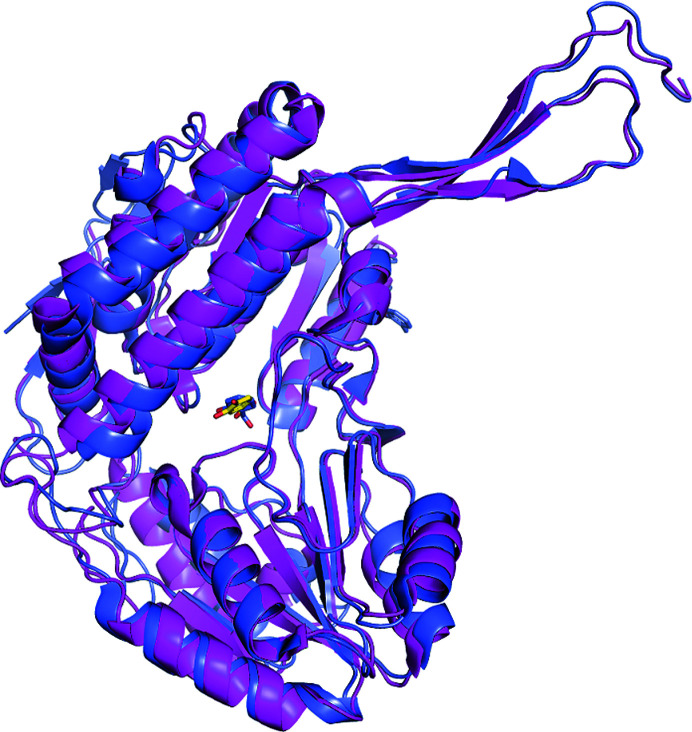
Superimposed monomers of the two salicylaldehyde dehydrogenases. SALD_AP_ is coloured magenta with the ligand in yellow sticks and NahF is coloured blue with the ligand in blue sticks.

It is not surprising that the best structural neighbour of SALD_AP_ is NahF (PDB entry 4jz6), which is the only salicyl­aldehyde dehydrogenase crystal structure that was available in the PDB prior to our crystal structure. The two crystal structures were superimposed (Fig. 7 ▸). Superimposition allows structural alignment of the residues and comparison of the substrate- and cofactor-binding sites. The high *Z*-score (Table 6 ▸) indicates high structural similarity between the two proteins, and superimposition/alignment of the structures further ascertained this similarity: 85% of the amino-acid residues structurally aligned well, with a root-mean-square deviation (r.m.s.d.) of 1.050 Å over 2580 equivalent atoms. The high *Z*-score and similar functional description indicate homology with possible implications for functional conservation. SALD_AP_ has 18 β-strands while NahF has 21. Conversely, NahF has 18 α-helices while SALD_AP_ has 20. In essence, these two proteins differ from each other at the N-terminus, where SALD_AP_ has a short N-terminal tail with only two β-strands. However, in addition to the β-strands possessed by SALD_AP_, NahF has three β-sheets at the N-terminus, making it an elongated version of SALD_AP_. This truncation of the N-terminus of SALD_AP_ might have happened during evolution as the region is located on the surface and makes no contact with other protein subunits. Hence, the region might not play a significant role in the protein. This finding strengthens the conclusion that proteins are evolutionarily more related by their structures than by their sequences. In favourable cases, structural similarity can reveal evolutionary connections that are difficult to detect using sequence comparisons.

The crystal structure that we have presented here will be useful in further studying the mechanisms of ligand binding (aldehydes/carboxylic acids) and catalysis in ALDHs. Also, the strategy we have reported serves as a proof of concept for the discovery and exploitation of novel enzymes from the environment. The detailed biochemical properties of recombinant SALD_AP_ will be published in a separate, future paper. The atomic coordinates and crystal structure of SALD_AP_ have been deposited in the Protein Data Bank with accession code 6qhn **.**

## Supplementary Material

PDB reference: metagenome-derived salicylaldehyde dehydrogenase from Alpine soil in complex with protocatechuic acid, 6qhn

## Figures and Tables

**Table 1:** Macromolecule-production information In the primers, the underlined sequences are the specific flanking sequences required to generate the overhangs necessary for ligation-independent cloning (LIC) of the gene into pLATE51 (p51) vector, which adds an N-­terminal 6×His tag to the target protein. The non-underlined sequences represent the SALD_AP_ gene-specific sequences.

Source organism	Metagenome
DNA source	Alpine paleosols
Forward primer	5′-GGTGATGATGATGACAAGAGGGGGCTCACCGTG-3′
Reverse primer	5′-GGAGATGGGAAGTCATTAAATGGGAAAGTGGCCG-3′
Expression vector	pLATE51 (p51)
Expression host	*E. coli* BL21 (DE3)
Complete amino-acid sequence of the construct produced	MRGLTVNFERINPMTNQTASTAKAMTAAEARAVADRAAAGFAGWSVLGPNARRAVLMKAAAALEARKDDFVQAMMAEIGATAGWAMFNLMLAASMIREAAALTTQIGGEVIPSDKPGCLALALREPVGVVLGIAPWNAPIILGVRAIAVPLACGNAVILKASEICPRTHGLIIESFAEAGFPEGVVNVVTNAPQDAGEVVGALIDHPAVKRINFTGSTGVGRIIAKRAAEHLKPCLLELGGKAPLVVLDDADLDEAAKAAAFGAFMNQGQICMSTERIIVVEAIAAEFTRRFAAKAQSMATGDPREGKTPLGAVVDRKTVDHVNTLIDDATAKGARIIAGGKGDSVLMSATVVDGVTAAMKLYRDESFGPIVGIIRAKDEADAVRLANDSEYGLAAAVFTRDTARGLRVARQIRSGICHINGPTVHDEAQMPFGGVGASGYGRFGGKAGIDQFTELRWITMETQPGHFPI

**Table 2:** Crystallization

Method	Sitting-drop vapour diffusion
Plate type	JCSG+
Temperature (K)	293
Protein concentration (mg ml^−1^)	12
Buffer composition of protein solution	20 m*M* sodium phosphate buffer, 20 m*M* NaCl pH 7.4, 2 m*M* TCEP
Composition of reservoir solution	0.1 *M* sodium acetate pH 4.6, 8%(*w*/*v*) PEG 8000
Volume and ratio of drop	100 + 100 nl drop
Volume of reservoir (µl)	40

**Table 3:** Data collection and processing Values in parentheses are for the highest resolution shell.

Diffraction source	Diamond Light Source
Wavelength (Å)	1.03865
Temperature (K)	100
Detector	PILATUS3 6M
Crystal-to-detector distance (mm)	336.60
Rotation range per image (°)	0.1
Total rotation range (°)	270
Exposure time per image (s)	0.020
Space group	*C*222_1_
*a*, *b*, *c* (Å)	116.8, 121.7, 318.0
α, β, γ (°)	90, 90, 90
Mosaicity (°)	0.069
Resolution range (Å)	27.8–1.90 (2.02–1.90)
Total No. of reflections	996150
No. of unique reflections	177080
Completeness (%)	99.8 (99.6)
Multiplicity	5.6 (5.7)
〈*I*/σ(*I*)〉	9.94 (1.09)
CC_1/2_ (%)	99.8 (55.5)
*R*_merge_ (%)	11.7 (124.1)
*R*_r.i.m._ (%)	12.9
Overall *B* factor from Wilson plot (Å^2^)	31.4

**Table 4:** Structure refinement Values in parentheses are for the highest resolution shell.

Resolution range (Å)	27.83–1.90
Completeness (%)	99.9
σ Cutoff	None
No. of reflections, working set	176985
No. of reflections, test set	8994
Final *R*_cryst_	0.177
Final *R*_free_	0.203
Cruickshank DPI	0.171
No. of non-H atoms	
Protein atoms	13636
Ligand	54
Water atoms	1597
Total	15287
R.m.s. deviations	
Bonds (Å)	0.010
Angles (°)	1.06
Average *B* factors (Å^2^)	
Overall	38.5
Protein	38.0
Ligand	39.5
Water	46.6
Ramachandran plot	
Favoured regions (%)	98.4
Additionally allowed (%)	1.6

**Table 5:** Thermal stability of SALD_AP_ showing its melting temperatures (*T*_m_) upon interaction with different ligands The *T*_m_ of SALD_AP_ bound to ligands was measured in the presence of 2 m*M* ligand and 1.5 m*M* NAD^+^. All experiments were carried out with 6 µ*M* enzyme. The values indicate the mean of triplicate measurements ± standard deviation. All results were compared with the *T*_m_ of the untreated enzyme (control) for statistical significance using one-way ANOVA and Dunnett’s multiple comparison post-test. * indicates a statistically significant difference (*p* < 0.05) between the test and the control.

Enzyme/ligand	Melting temperature (*T*_m_) (°C)
Untreated	67.9 ± 0.17
NAD	68.7 ± 0.25
Protocatechuic acid	69.2 ± 0.25*
Salicylaldehyde	67.7 ± 0.29
NAD + salicylaldehyde	70.3 ± 0.35*

**Table 6:** Structural neighbours of novel SALD_AP_

Rank	PDB code, chain	*Z*-score	R.m.s.d. (Å)	No. of aligned residues	No. of residues	Identity (%)	PDB description
1	4jz6, *A*	54.4	1.4	466	484	48	Salicylaldehyde dehydrogenase (NahF)
2	3prl, *D*	50.8	2.4	458	480	33	NADP-dependent glyceraldehyde-3-phosphate dehydrogenase
3	3efv, *A*	50.8	1.8	451	459	33	Putative succinate-semialdehyde dehydrogenase
4	3vz0, *A*	50.1	1.8	449	456	28	Putative NAD-dependent aldehyde dehydrogenase
5	4nmk, *C*	50.1	1.5	463	490	33	Aldehyde dehydrogenase
6	3pqa, *B*	49.9	1.8	451	458	31	Lactaldehyde dehydrogenase
7	3jz4, *A*	49.9	1.5	455	481	34	Succinate-semialdehyde dehydrogenase (NADP^+^)
8	3ek1, *A*	49.8	1.5	457	485	32	Aldehyde dehydrogenase
9	5x5t, *A*	49.7	1.6	455	476	32	α-Ketoglutaric semialdehyde dehydrogenase
10	1euh, *A*	49.4	1.4	454	474	34	NADP-dependent aldehyde dehydrogenase
